# New uses of the Migraine Screen Questionnaire (MS-Q): validation in the Primary Care setting and ability to detect hidden migraine. MS-Q in Primary Care

**DOI:** 10.1186/1471-2377-10-39

**Published:** 2010-06-08

**Authors:** Miguel J Láinez, Jesús Castillo, Manuel Domínguez, Gemma Palacios, Silvia Díaz, Javier Rejas

**Affiliations:** 1 Department of Neurology, University Hospital of Valencia, Valencia, Spain; 2 Primary Care Health Center of Camargo, Muriedas, Cantabria, Spain; 3 Department of Neurology, Hospital Central de la Defensa, Madrid, Spain; 4 CNS Therapeutic Area, Medical Unit, Pfizer SA, Alcobendas (Madrid), Spain; 5 Health Outcomes Research, Medical Unit, Pfizer Spain, Madrid, Spain

## Abstract

**Background:**

PC plays an important role in early diagnosis of health disorders, particularly migraine, due to the financial impact of this disease for the society and its impact on patients' quality of life. The aim of the study was to validate the self-administered MS-Q questionnaire for detection of hidden migraine in the field of primary care (PC), and to explore its use in this setting.

**Methods:**

Cross-sectional, observational, and multicentre study in subjects above 18 years of age patients attending PC centers (regardless of the reason for consultation). A MS-Q score ≥ 4 was considered possible migraine. Level of agreement with IHS criteria clinical diagnosis (kappa coefficient), and instrument's validity properties: sensitivity, specificity, positive (PPV) and negative (NPV) predictive values were determined. The ability of the instrument to identify possible new cases of migraine was calculated, as well as the ratio of hidden disease compared to the ratio obtained by IHS criteria.

**Results:**

A total of 9,670 patients were included [48.9 ± 17.2 years (mean ± SD); 61.9% women], from 410 PC centers representative of the whole national territory. The clinical prevalence of migraine according to the IHS criteria was 24.7%, and 20.4% according to MS-Q: Kappa index of agreement 0.82 (p < 0.05). MS-Q sensitivity was 0.82 (95% CI, 0.81 - 0.84), specificity 0.97 (95% CI, 0.98 - 0.99), PPV 0.95 (95% CI, 0.94 - 0.96), and NPV 0.94 (95% CI, 0.93 - 0.95). No statistically significant differences were found in the percentages of patients with *de novo *and hidden migraine identified by MS-Q and by IHS criteria: 5.7% vs. 6.1% and 26.6% vs. 24.1%, respectively.

**Conclusions:**

The results of the present study confirm the usefulness of the MS-Q questionnaire for the early detection and assessment of migraine in PC settings, and its ability to detect hidden migraine.

## Introduction

Migraine is a highly disabling disease with a prevalence of about 10-12% in the general population. The risk of experiencing migraine increases from the age of 16 years, being 2 to 3-fold more frequent in women compared to men [1]. In Spain, 14.1% of patients attending outpatient neurology clinics are diagnosed of migraine and, when referred to subjects less than 65 years of age, this percentage attains 20.7% [2].

On the other hand, primary care (PC) physicians play an essential role in the diagnosis and management of migraine; over 90% of migraine patients attending outpatient neurology clinics are referred from their family doctors [3]. Furthermore, recent data have shown a significant increase of the number of PC visits due to migraine, from 9.4 visits per 1.000 patients in 1990, to 18 visits per 1.000 patients in 1998 [4], this involving an important assistance load. However, migraine continues being an underdiagnosed condition. Calculations indicate that barely 50% of these patients visit their doctor trying to find help [5] and, in many cases, a proper diagnosis and treatment may take years [6-8]. In order to properly establish a diagnosis of migraine, it is essential to know the IHS classification criteria and apply these criteria in clinical practice [9]. However, a recent survey conducted in 721 Spanish PC doctors, indicated that only half of them knew this classification, and only 5.4% were using this classification in daily clinical practice. Although this lack of knowledge, doctors stated they were interested in migraine and, compared to the mean time per visit for other diseases, were devoting a longer time to migraine patients [10].

Bearing in mind the impact of migraine on patients' quality of life [11,12], and its socio-economic consequences [13,14], an early approach to the disease is becoming increasingly necessary. With this purpose and, in accordance to recommendations of experts on management of migraine [7,15,16], instruments for early detection have been developed in recent years, as the Diagnostic Headache Diary [17], the UCSD Migraine Questionnaire [18], the ID Migraine [19], the Brief Headache Screen [20], the 3-Question Headache Screen [21], and other [22-26], with the aim of assisting PC physicians to identify migraine patients in the shortest possible time, due to the important assistance load of health centers. This would not only improve the clinical approach to the diagnosis of migraine, but also contribute to improve patients' awareness of their disease, as often patients are not aware of this disorder and of its social, occupational, and clinical implications [27,28].

**Figure 1:**
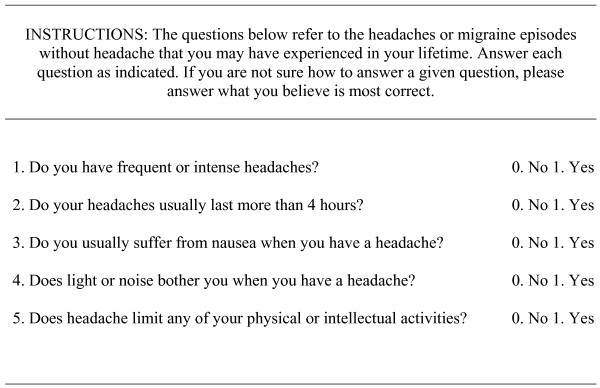
**Migraine Screen — Questionnaire (MS-Q)**.

Of the previously mentioned instruments, only ID Migraine and 3-Question Headache Screen have demonstrated their clinical usefulness, although the specificity of the first one [19] and the sensitivity of the second one [21] to identify migraine are low, this limiting their practical usefulness. In addition, these instruments have not been validated yet for the Spanish population. In 2005, a new instrument developed by a group of experts, based on the International Headache Society (IHS) criteria, and fulfilling the principles of medicine based on evidence, was validated [29]. This is a self-administered questionnaire for the screening of migraine, known as MS-Q, Migraine Screen Questionnaire that may be used both, in clinical practice and research projects (see Figure 1). MS-Q is rapidly executed by patients, and contributes to easily identify symptoms suspicious of migraine for a later medical confirmation of the diagnosis. This renders MS-Q a new instrument able to optimize the management of migraine patients with an important saving of time.

Thus, it was reasonable to conduct the present study, one of which primary objectives was the validation of MS-Q questionnaire as an instrument for migraine detection in daily PC medical practice, not previously validated, as well as to determine the prevalence of migraine and the ratio of hidden migraine.

## Materials and methods

### Study Design

A cross-sectional, observational, and multicentre study was designed. Patients of both genders over 18 years of age who attended PC centers for any reason were recruited, and informed consent was obtained. Participant centers were randomly selected by regional quotes to preserve the national representative nature according to regional population weights. However, patients were consecutively recruited. Patients unable to complete questionnaires or to understand the study consent were excluded. Due to the observational nature of the study design, the investigator decision on the most appropriate treatment for a particular patient was never interfered. The conduct of the study should not involve additional risks, complementary tests, or additional visits relative to the management of migraine patients in daily clinical practice. The study was approved by the IRB of the University Hospital of Valencia and was conducted in accordance to the Helsinki declaration for research in the human being.

### Sample Size

As previously mentioned, it was estimated that in the general population, the prevalence of migraine is differently distributed depending on gender. For this reason, to estimate the specific prevalence of migraine by gender, it was considered appropriate to take into account this aspect. A previous study indicated 18.2% prevalence of migraine among women, and 6.5% among men [30]. Taking women's prevalence for sample size calculation, as this is the highest value and requires a higher sample size (in order to obtain an adequate sample to accurately estimate prevalence in both genders), a sample of 5,709 subjects was estimated to provide an accuracy of ± 1% for the calculation of prevalence of migraine in women with a 95% confidence interval.

The present study was foreseen to be conducted in the PC setting and, as in accordance with 1999 data from the National Statistics Institute (INE) [31], 54.21% of subjects who attend primary care and specialized centers, and medical clinics were female patients, the 5,709 women needed to estimate prevalence of migraine would represent 54.21% of the total sample to be recruited. Thus, a total sample of 10,532 patients was estimated. With an anticipated loss for the analysis of 5% of patients, a total sample of 11,087 patients was to be recruited. The PASS package, 2002 version was used for calculations.

### Assessment Measures for the Diagnosis of Migraine

Patients fulfilling inclusion and exclusion criteria completed the self-administered questionnaire MS-Q for the screening of migraine [29]. This questionnaire consists on 5 questions related to frequency and characteristics of headache, as well as to the presence or absence of migraine related symptoms. A score of 0 was obtained for each negative answer (NO), and of 1 for each positive answer (YES). A cutting-point indicating suspicion of migraine was established at ≥ 4 points, while an <4 score indicated no suspicion of migraine [29].

Regardless MS-Q results, doctors checked their suspicion of a diagnosis of migraine according to their clinical judgment and IHS diagnostic criteria [9]. The (diagnosis of migraine according to clinical judgment- IHS) was recorded on the case report form (CRF) with the following question: *(In accordance to your clinical judgment and to the recent IHS criteria for the diagnosis of migraine (International Diagnosis of Migraine Criteria), does the patient currently present a diagnosis of migraine?) *with the following possible answers: (yes or no). All participant doctors received training on IHS migraine criteria, and a contact phone number was set up to clarify possible questions about the study.

### Data Analysis

In order to guarantee the project's quality, clinical data were validated executing computer filters based on validation rules, to identify missing values or inconsistencies of clinical data relative to the case report form (CRF) and the protocol. After review, inconsistent data were modified in the database according to the CRF, provided possible inconsistencies had emerged from errors generated during data tabulation process. At any time, the reliability and rigor of the analysis was guaranteed. The SPSS^®^, version 12.0.1 statistical program was used for all statistical analysis.

Data were descriptively analyzed, mean, standard deviation, and 95% confidence intervals calculated for quantitative variables, and frequencies for qualitative variables, in the overall and by gender samples. Patients socio-demographic and clinical variables: age, gender, family past history of migraine (yes/no), previous diagnosis of migraine (yes/no), type of migraine, type of health center where patient's migraine was being treated, and the reason for the current visit were collected. Thereafter, and according to the study primary objective, prevalence of suspected diagnosis of migraine according to MS-Q, with the corresponding two-way 95% confidence interval, was determined both, for the overall and by gender samples. Subsequently, based on the results obtained for the MS-Q questionnaire and according to clinical judgment/IHS, concordance in the presence or absence of diagnosis of migraine suspicion was analyzed by kappa index calculation. The following ranges are recommended for interpretation of the Kappa index (Landis) [32]: 0.40-0.59 (moderate concordance); 0.60-0.79 (substantial concordance), and ≥ 0.8 (excellent concordance). Additionally, the following indicators of validity and safety of the questionnaire as a test to detect migraine were estimated: sensitivity, specificity, positive predictive value (PPV), negative predictive value (NPV), positive probability ratio (PPR), and negative probability ratio (NPR).

In some cases, data on fulfillment of IHS migraine diagnosis criteria were not reported or were missing. However, when in other CRF sections migraine was specified as a concomitant disease, the patient received treatment with the indication of (migraine), the reason for visit was (migraine), or the patient had a previous diagnosis of migraine, a diagnosis of migraine according to clinical judgment/IHS was considered for that patient. In case of data inconsistency relative to clinical diagnosis of migraine (inconsistency between diagnosis data and other CRF data), the analysis of diagnosis of migraine concordance between MS-Q questionnaire and clinical judgment/IHS was repeated after excluding these data from the analysis (analysis with refined IHS criteria).

The percentage of patients with a *de novo *PC diagnosis by MS-Q was calculated, i.e., the percentage of patients with no previous diagnosis of migraine who indicated a total scores ≥ 4 points in the questionnaire during the visit, and the percentage of patients with *de novo *diagnosis in accordance to clinical judgment/IHS. Last, the percentage of (hidden migraine) was calculated, i.e., percentage of patients with *de novo *diagnosis vs. total number of migraine patients diagnosed by both methods (clinical judgment/IHS, and MS-Q questionnaire).

In case of normal distribution, comparisons were made by a Student T test for independent samples (Gauss adjustment by a Kolmogorov-Smirnov test), by a Mann-Whitney U test for non-normal quantitative variables, and by a Chi-square test for nominal variables. A two-way p value < 0.05 was considered significant.

## Results

### Description of the Overall Valuable Sample

A total of 410 investigators from 371 PC health centers representative of the whole national territory recruited a total sample of 9,670 patients of which 324 patients (3.4%) not fulfilling any of the inclusion criteria were excluded: 1% were under 18 years of age, 0.54% were unable to complete questionnaires or understand the study informed consent, and for 1.8% of patients data were not sufficient to estimate the study primary variable (MS-Q questionnaire). In the total valuable sample (n = 9.346), 61.9% were female patients with a mean age of 48.9 years (SD: 17.2), and the largest part were over 40 years (65%). Male patients mean age was higher: 50.1 (SD: 17.2) vs. 48.2 (17.2) years, p < 0.001.

A 26.3% of patients indicated a family past history of migraine, and 21.1% had a previous diagnosis of migraine. Among these patients, the most common type of migraine was without aura (41.4%), and most patients attended PC centers for disease management (74.1%, Table 1). Reasons for consultation were the following: administrative reason, prescription or work leave (18.2%), review (10.6%), and disease (56.3%). Neurological diseases represented the third reason for consultation (8.6%), being migraine the most frequent: 85.9% (Table 2).

**Table 1:** Background of migraine in overall and by gender samples

	Male (%)	Female (%)	Overall (%)	p^1^
**Family past history of migraine**				
Yes	19.6	30.5	26.3	<0.001
**Previous diagnosis of migraine?**				
Yes	12.4	26.3	21.0	<0.001
**Type of migraine**^(2)^				
With aura	32.5	32.8	32.7	0.010
Without aura	35.3	43.1	41.4	
With and without aura	27.5	20.2	21.8	
Other	4.8	4.0	4.1	
**Where is your migraine being controlled?**				
Primary care	77.9	73.1	74.1	
Neurology	10.2	12.3	11.8	
Other	3.9	3.6	3.7	0.527
Primary care and Neurology	7.8	10.4	9.8	
Primary care and other	0.0	0.2	0.2	
Neurology and other	0.0	0.2	0.1	
Primary care, Neurology and other	0.3	0.3	0.3	

^1^Chi-square test

**Table 2:** Reasons for the present visit: diseases or other related reasons.

Reason for visit	Number of patients^2^	Percentage^3^
**Diseases**	**5,259**	**56.3**
Muscle -- skeletal diseases	1,182	12.6
Respiratory diseases	899	9.6
Neurological diseases^1^	803	8.6
Cardiovascular diseases	654	7.0
Gastrointestinal -- Hepatic diseases	361	3.9
Uro-gynecological diseases	290	3.1
Various signs and Symptoms	270	2.9
Psychiatric diseases	268	2.9
Endocrine -- Metabolic diseases	264	2.8
Dermatologic diseases	251	2.7
Organs of senses diseases	236	2.5
Infectious diseases	27	0.3
Malignancies	17	0.2
**Other disease-related reasons**	**2,654**	**28.4**
Administrative -- Prescriptions -- Work leaves	1,703	18.2
Review --Routine control	994	10.6

^1^690 of these patients with migraine (85.93%); ^2^A patient could simultaneously have > one reason; ^3^Percentages calculated from overall evaluated patients.

### Prevalence of Migraine

#### Diagnosis of migraine suspicion according to MS-Q questionnaire

MS-Q questionnaire mean total score was 1.6 (1.8) for the overall sample: 1.0 (1.5) for men, and 1.9 (1.9) for women, p < 0.001. Significant differences in the percentage of affirmative answers of all questionnaire items were observed depending on gender, with higher frequencies found among women (p < 0.05). MS-Q questionnaire total score was ≥ 4, the cutting-point for migraine suspicion, in 20.4% (95% CI: 19.5% - 21.2%) of patients. Statistically significant differences were observed depending on gender, and scores > 4 were more frequent among female patients: 26.2% (25.1% - 27.3%) for women vs. 11.0% (10.0%-12.0%) for men; p < 0,001.

#### Diagnosis of migraine according to clinical judgment -IHS

The percentage of patients with a diagnosis of migraine according to clinical judgment-IHS at the time of visit was 24.7% (23.7% - 25.6%). A higher prevalence of migraine was observed among women compared to men: 31.0% (29.7% - 32.2%) vs. 14.3% (13.1% - 15.5%) respectively; p < 0,001.

#### Validity of MS-Q for the suspicion of migraine diagnosis

Results of MS-Q questionnaire validity as a screening test for migraine, using the clinical judgment-IHS as the reference diagnosis are shown on Table 3. Cohen Kappa coefficient of agreement, and sensitivity, specificity, PPV, NPV, PPR, and NPR indicators are shown for the original analysis and the refined analysis (after exclusion of patients with inconsistent data). In the original analysis the Kappa coefficient was 0.82, and 0.84 in the refined analysis, this indicating an excellent concordance between the two analyzed variables. Sensitivity, specificity, PPV, NPV, PPR, and NPR indicators obtained in the refined analysis were equal or higher than those obtained in the original analysis. Minimum MS-Q validity index, i.e., for the original analysis, were the following: sensitivity = 0.82, specificity = 0.97, PPV = 0.90, NPV = 0.94%, PPR = 27.66 y NRP = 0.18.

**Table 3:** MS-Q questionnaire validity properties being the diagnosis of reference that obtained according to clinical judgment -IHS in overall and by gender samples.

In the overall sample, according to IHS criteria	Validity of MS-Q questionnaire						
	
	Kappa	Sensitivity	Specificity	PPV	NPV	PPR	NPR
Overall							
Original database (N = 8.073)	0.82*	0.82 (0.81 - 0.84)	0.97 (0.97 - 0.97)	0.90 (0.89 - 0.91)	0.94 (0.94 - 0.95)	27.66 (23.93 - 31.97)	0.18 (0.17 - 0.20)
Depurated database (N = 7.528)	0.84*	0.82 (0.81 -- 0.84)	0.98 (0.98 - 0.99)	0.95 (0.94 -- 0.96)	0.94 (0.93 - 0.95)	49.00 (40.02 -- 60.01)	0.18 (0.16 - 0.20)

Gender							
Male (N = 3.076)	0.79*	0.77 (0.73 - 0.81)	0.98 (0.98 - 0.99)	0.88 (0.85 - 0.91)	0.96 (0.94 --0.95)	44.08 (32.85 -- 59.16)	0.24 (0.20 -- 0.28)
Female (N = 4.997)	0.82*	0.84 (0.82 -0.86)	0.96 (0.95 - 0.97)	0.91 (0.89 - 0.92)	0.94 (0.93 - 0.95)	21.32 (18.06 - 25.17)	0.17 (0.15 - 0.19)

PPV: Positive Predictive Value; NPV: Negative Predictive Value; PPR: Positive Probability Ratio; NPR: Negative Probability Ratio. * p < 0.001

The analysis by gender of these indicators indicated slightly higher MS-Q Kappa index and sensitivity values in the female group, while specificity, PPR, and NPR values where higher in the male group (table 3).

### Diagnosis of De Novo and Hidden Migraine

The mean percentages of patients with suspicious *de novo *diagnosis were 5.7% by MS-Q, and 6.1% by clinical judgment-ICH, and no statistically significant differences were found between both diagnostic methods (Table 4). With both methods, the women group indicated a significant higher percentage of *de novo *diagnosis compared to the men group.

**Table 4:** Percentage of patients with *de novo *and hidden migraine in overall and by gender samples.

Percentage of patients^1^	Population	MS-Q questionnaire	Clinical judgment - IHS	Difference
Diagnosis *de novo*^2^	Overall sample	5.7% (5.2% - 6.2%)	6.1% (5.5% - 6.6%)	3.1% (-1.1% - +3.7%)
	Male	3.4% (2.8% - 4.0%)	3.7% (3.0% - 4.4%)	3.3% (-1.2% - +5.9%)
	Female	7.2% (6.5% - 7.9%)	7.5% (6.8% - 8.2%)	3.2% (-1.3% - +6.9%)
Hidden migraine	Overall sample	26.6% (24.7% - 28.6%)	24.1% (21.4% - 29.6%)	1.4% (-1.4% - +4.2%)
	Male	29.4% (24.8% - 33.9%)	25.5% (21.4% - 29.6%)	2.9% (-2.3% - +10.0%)
	Female	26.0% (23.8% - 28.3%)	23.9% (21.8% - 26.1%)	2.1% (-1.0% - +5.2%)

^1 ^Percentages from the number of valid cases were calculated (data not reported in some cases). ^2 ^Statistically significant differences were observed depending on the gender group (p < 0.001; Chi-square test).

No statistically significant differences were found between percentages of hidden migraine identified by both methods. Mean percentages of hidden migraine were 24.0% (clinical judgment-IHS), and 26.6% (MS-Q questionnaire). For this variable, the mean percentage of hidden migraine was higher among men compared to women. However, these differences were not statistically significant: 29.4% (24.8% - 33.9%) vs. 26.0% (23.8% - 28.3%) with MS-Q questionnaire, and 25.5% (21.4% - 29.6%) vs. 23.9% (21.8% - 26.1%) with IHS criteria (Table 4).

## Discussion

As anticipated, the observed prevalence of migraine, 24.7% by clinical judgment-IHS, and 20.4% by MS-Q questionnaire, was higher than the prevalence reported in studies conducted in general population samples [33-37], as non-selected patients attending PC centers were included. 5.7% of the patients attending a visit had a MS-Q diagnosis of *de novo *migraine. This represents 26.6% of hidden migraine when a *de novo *diagnosis is referred to the migrainous population recruited for the study, and confirms the usefulness of this questionnaire in common medical practice. These data are very similar to those obtained by the clinical diagnosis in accordance to IHS criteria in the same population: 6.1% and 24,1%, respectively. Regarding differences by gender, prevalence of migraine in women was higher compared to men, consistently with other epidemiologic studies [5,37-40], as well as percentages of female patients with *de novo*, and hidden migraine diagnoses.

In this study, the percentage of hidden migraine revealed the magnitude of the problem of underdiagnosis among migrainous patients and, as previously mentioned, a proper diagnosis and treatment may take several years [3,6-8]. A study conducted in neurology clinics indicated that 23.9% of migraine patients referred form PC were not receiving any type of treatment, while among patients attending neurology clinics without a previous PC diagnostic approach was 85.7% [3]. This data indicate the importance of the diagnosis when a patient has to initiate treatment, bearing in mind the level of disability both, in work and day life activities, this disorder produces [14,35].

On the other hand, as already observed in other previous studies of our sanitary area [41], migraine was found to be the primary neurological disease evaluated in PC centers, representing up to 85.93% of consultations for neurological disorders, and most patients were managed in PC centers (74.1% of patients vs. 11.8% managed in neurology specialist centers). It is important to underline the role of the PC physician for the detection and classification of patients with headache, in order to directly treat them at the PC level, or to refer these patients to a specialized level when a headache of uncertain origin or of a more serious nature than migraine is suspected [7,42]

In addition, the study tested a new use of the instrument, its implementation at primary care level. MS-Q indicated to be an easy and simple to implement instrument for early detection of migraine in PC, and its implementation should result in a more appropriate diagnostic and therapeutic management of patients and, thus, a lower load for the society and patients. These statements are based on the concordance with the IHS criteria clinical diagnosis, determined by the Kappa index, shown in the validation of the MS-Q questionnaire in PC, that can be considered good, with a range of 0.82 to 0.84 depending on the sample used, original or refined sample. Nevertheless, both values are higher than the Landis criteria recommended value of 0.7 [32]. In this sense, the similarity between the results observed in a non-selected population attending PC centers are those observed during instrument's development, which included working and neurological clinics populations, is to be underlined. However, in the PC setting, the instrument indicated to be more specific, 97% specificity, but less sensitive, 82% sensitivity, although within the appropriate limits for a screening test [29,43]. Other psychometric properties of the analysis were almost optimal, with PPV and NPV ≥ 90%.

On the other hand, the comparison between MS-Q and other recent and similar questionnaires for the screening of migraine (ID Migraine [19] y Brief Headache Screen [20]) psychometric properties, confirmed that MS-Q questionnaire psychometric properties are more appropriate for a screening instrument. The instrument indicated a specificity higher than the other (0.75 and 0.93, respectively), and its sensitivity was higher than that of ID Migraine (0.75), and Brief Headache Screen (0.78) when used in chronic migraine patients. MS-Q also indicated a greater sensitivity than that observed for the 3-Question Headache Screen questionnaire [21], which properly classified 77% of the 3.014 migraine patients included in the instrument's validation study.

The present study, however, presents some limitations. First, the test-retest reliability assessment of the questionnaire is still pending; due to the important difficulties found in common medical practice. In a non-selected population, MS-Q seemed to slightly underestimate the actual prevalence of migraine using clinical judgment --IHS in this setting, although showed good indicators in accordance with the mentioned criteria. In addition, in contrast with Brief Headache Screen, MS-Q could not discriminate between patients with chronic and episodic migraine. On the other hand, this instrument was validated in Spanish from Spain, and might need subsequent validations before being used in other Spanish contexts. Last, due to the magnitude of patients recruitment in our study, we did not assess the incidence of incorrect diagnoses as, due to the possibility of erroneous migraine diagnosis, an expert panel would had been necessary to review patients' diagnoses [8].

Any research focusing the early detection of this disease will be welcome; the amount of years migraine patients spend without a proper diagnosis and treatment is unacceptable. In addition, it is essential that patients become conscious of their disease, as this is the first step to request medical care, receive treatment, as well as to follow day life recommendations to improve their quality of life and have positive consequences on their social and professional areas. Finally, because migraine may generally be identified early in life and is associated with multiple co-morbidities, that effective screening tool can define migraine and a population with other potential health risks to the primary care provider. Then, using migraine screening as a sentinel sign of other possible health conditions.

## Conclusions

In conclusion, although the previously mentioned limitations, it can be stated that, based on the results of the present study, the MS-Q questionnaire is a useful instrument for early detection and assessment of migraine also in the PC setting as, in addition of its good psychometric properties, only consists on five questions, resulting in the most feasible of the questionnaires used in this setting, and may save a very valuable time in PC centers.

## Abbreviations

IHS: International Headache Society; MS-Q: Migraine Screen Questionnaire; PC: Primary Care; NPV: negative predictive value; PPV: positive predictive value; PPR: positive probability ratio; NPR: negative probability ratio.

## Competing interests

This study has been funded by an unrestricted grant from Pfizer Spain. Javier Rejas and Silvia Díaz are full-time employees at Pfizer Spain. Gemma Palacios was employed at Pfizer Spain at the time of conducting the study but currently is not working at Pfizer. The other authors have not any conflict of interest with the source of funding of the study.

## Authors' contributions

MJL, JC and MD participated in the design of this study, interpretation of data and the writing of this manuscript. GP and JR were responsible of the idea of the study, participated in the design and preparation of the manuscript. SD participated in the analysis of data and in the preparation of the manuscript. All of the authors approved the content of the manuscript.

## Pre-publication history

The pre-publication history for this paper can be accessed here:

http://www.biomedcentral.com/1471-2377/10/39/prepub
